# Food Neophobia and Two Facets of Orthorexia Among Women: Cross-Sectional Study

**DOI:** 10.3390/bs15010070

**Published:** 2025-01-15

**Authors:** Tuba Yalçın, Seda Çiftçi, Elif Esra Ozturk

**Affiliations:** 1 Department of Nutrition and Dietetics, Faculty of Health Sciences, Izmir Katip Çelebi University, 35620 Izmir, Türkiye; 2 Department of Nutrition and Dietetics, Faculty of Health Sciences, Izmir Democracy University, 35140 Izmir, Türkiye; seda.ciftci@idu.edu.tr; 3 Department of Gastronomy and Culinary Arts, Faculty of Fine Arts and Architecture, Gaziantep Islam Science and Technology University, 27260 Gaziantep, Türkiye; elifesra.ozturk@gibtu.edu.tr

**Keywords:** orthorexia nervosa, healthy orthorexia, food neophobia, women

## Abstract

The purpose of this study was to investigate the link between food neophobia and two dimensions of orthorexia in women. This cross-sectional study of 985 women aged 18 years and over was conducted using face-to-face questionnaires. Women who had a disability, had a chronic disease, or were pregnant or breastfeeding were excluded. Participants provided information on their sociodemographic details (age and educational level) and frequency of physical activity. Orthorexic tendencies were assessed using the Teruel Orthorexia Scale. The women’s attitude towards trying new foods was assessed using the Food Neophobia Scale. A total of 337 participants (34.2%) were neophilic, 322 participants (32.7%) were neutral, and 326 participants (33.1%) were neophobic. There was no correlation between food neophobia scores and either age or body mass index. However, food neophobia was positively correlated with healthy orthorexia and orthorexia nervosa (*p* < 0.05). The mean individual scores for orthorexia nervosa and healthy orthorexia according to the Teruel Orthorexia Scale were 11.45 ± 3.91 and 20.04 ± 4.31, respectively. The results indicate that individuals with orthorexia nervosa have higher food neophobia scores, reflecting a greater reluctance to try unfamiliar foods, whereas individuals with healthy orthorexia do not show significant differences in food neophobia tendencies. This distinction highlights the importance of distinguishing between pathological and non-pathological eating behaviors when addressing dietary concerns.

## 1. Introduction

Food neophobia (FN) is characterized by a reluctance or refusal to eat novel or unfamiliar foods (Białek-Dratwa et al., 2022). Evidence suggests that FN may be negatively associated with the acceptance of not only novel/foreign foods but also familiar foods (Karaağaç & Bellikci-Koyu, 2023). Although it is not formally classified as a disorder in the Diagnostic and Statistical Manual of Mental Disorders (DSM-5-TR) (Association, 2023), it is recognized as an important psychological and behavioral phenomenon (Barrena & Sánchez, 2013). FN is typically measured using the Food Neophobia Scale (FNS), which assesses an individual’s tendency to avoid novel foods (Pliner & Hobden, 1992). A study conducted in Türkiye found a prevalence of FN of14.6%, while 15.9% of university students were found to be neophilic (Basaran & Ozbek, 2023). Another study found that 22.8% of participants were neophilic and 24.2% were neophobic among Turkish adults (Çakır et al., 2023). The prevalence of FN in adults varies, with estimates suggesting that a significant minority retain neophobic tendencies, which may affect their dietary diversity and nutrient intake (Roehr, 2013).

Orthorexia nervosa (OrNe) was first conceptualized in 1997 by family physician Steven Bratman (Bratman, 1997). The term is derived from the Greek words orthos, meaning ‘correct’, and orexis, meaning ‘appetite’, and was introduced to describe an obsessive preoccupation with ‘correct’ eating that he observed in his patients. In 2016, Bratman and Dunn further refined the definition of OrNe, distinguishing it from a general desire to live a healthy life by highlighting its association with negative outcomes, including malnutrition and impaired social functioning (Dunn & Bratman, 2016). Some people may pathologize their ideas and interest in healthy food preferences. This can lead to an obsession with consuming only what they think is healthy (WHO, 2020). Studies have reported that individuals with high orthorexic tendencies often exhibit strict dietary rules and obsessive food-related behaviors, which can lead to significant disruptions in their daily lives, including social and psychological difficulties (Oberle et al., 2017; Varga et al., 2014). According to the literature, orthorexic behavior can be characterized either as a reasonable concern in proper nutrition or as a compulsive fixation on healthy eating. Studies indicate that it is difficult to distinguish between OrNe and non-pathological attitudes towards healthy eating (Barrada & Roncero, 2018; Şentürk et al., 2022). For this purpose, a new term, “healthy orthorexia (HeOr)”, has recently emerged in the literature (Barthels et al., 2019; Şentürk et al., 2022). The ORTO-15, the most widely used self-report measure for the assessment of OrNe, has shown an unstable factorial structure in diverse populations. It has been criticized for its inadequate psychometric properties and inability to effectively discriminate between healthy and pathological eating behaviors (Cena et al., 2019; Valente et al., 2019). A relatively recent development in this area is the Teruel Orthorexia Scale (TOS), which was designed to assess both HeOr and OrNe (Barrada & Roncero, 2018). Therefore, orthorexia can be divided into two distinct dimensions. HeOr refers to a constructive interest in dieting and self-assessed healthy behaviors related to eating patterns (Barrada & Roncero, 2018). Conversely, OrNe is characterized by an intense fixation on eating healthy foods, which may lead to contradictory concerns when trying to adhere to this goal, as well as distress when deviating from prescribed eating behaviors (Depa et al., 2019). However, HeOr scores are not necessarily indicative of actual healthy eating patterns and should not be equated with them. Individuals who score high on the HeOr may place great importance on a healthy diet and make serious efforts to adhere to it. Healthy eating patterns may not correspond to a precise definition. Therefore, it is important to avoid using the two terms interchangeably, and a high score on the HeOr does not indicate that an individual has a truly healthy diet (Roncero et al., 2021).

Understanding FN is crucial in contexts such as orthorexia, a behavior characterized by an obsession with healthy eating to the point of restrictive dietary patterns (Roehr, 2013). Individuals with orthorexia are considered a high-risk group for nutrient deficiencies due to their adoption of restrictive diets that significantly reduce the diversity of essential nutrients (Horovitz & Argyrides, 2023). A study suggests an association between FN and OrNe, as individuals with higher FNS scores may be more prone to restrictive eating behaviors associated with OrNe (Uçar et al., 2021). Exploring this relationship helps to elucidate the psychological underpinnings of eating behaviors and provides insights into how food-related anxiety contributes to the development and maintenance of disordered eating patterns. The existing literature has mainly focused on the pathological manifestation of OrNe in research studies (Barthels et al., 2019). Only a few studies have investigated the two-dimensional aspect of orthorexia, which distinguishes between a healthy interest in eating (HeOr) and a pathological fixation on eating healthy foods (OrNe) (Awad et al., 2022; Barthels et al., 2019).

Research suggests that physical activity can be both positively and negatively associated with orthorexic tendencies. For instance, while moderate physical activity is often associated with general health awareness and constructive dietary practices, excessive exercise has been linked to increased orthorexic behaviors, as individuals may seek to supplement restrictive eating habits with compulsive exercise to achieve perceived health ideals (Oberle et al., 2018; Strahler et al., 2021). Similarly, physical activity may influence FN by either by promoting dietary diversity through exposure to diverse nutritional requirements or by reinforcing neophobic tendencies when paired with rigid dietary regimens (Guzek et al., 2018). Including physical activity as a variable in the context of OrNe and FN provides valuable insights into their interplay with lifestyle behaviors.

Furthermore, the relationship between OrNe and gender remains inconclusive in the existing literature. While some studies report that orthorexia nervosa may affect women more often than men (Elias et al., 2022; Parra-Fernández et al., 2018; Sanlier et al., 2016), other studies show that there is no gender difference in orthorexia nervosa (Luck-Sikorski et al., 2019; Oberle et al., 2017). Women may influence the food choices and diet quality of family members within the household (Fulkerson et al., 2008). The relationship between OrNe and FN, among women, remains poorly understood. Therefore, further research is needed to understand the link between orthorexia and FN in relation to both sub-dimensions of orthorexia in women. Therefore, investigating the association between ON and FN in women is of great societal importance. Therefore, the purpose of this cross-sectional study is to determine the relationship between FN and two sub-dimensions of orthorexia in women.

## 2. Materials and Methods

### 2.1. Participants and Procedure

This cross-sectional study was conducted in Izmir, Türkiye, between January and June 2023. The researchers reached a total of 985 women living in the central districts of Izmir using the random sampling method. They were randomly selected in various public places such as universities, parks, and shopping malls to complete a face-to-face questionnaire, no monetary or any other benefit was offered to them, and their participation was completely voluntary. Participants were given a clear explanation of the purpose of the study, and those who wished to participate were included. The inclusion criteria were women aged 18 years or older, living in the İzmir region, who agreed to participate in the survey and who were able to read, understand, and write in Turkish. Women who had any disability, were suffering from any chronic disease, or were pregnant or breastfeeding were excluded.

We enrolled women in our study for several reasons. First, there is evidence suggesting that OrNe may be more common in women than in men. Women may also influence the food choices and diet quality of family members in the household. However, the relationship between orthorexia and FN specifically in women remains unclear. Therefore, the identification of orthorexia and FN, especially in women, is very important for society. Additionally, Freco et al. highlighted the intricate differences in dietary patterns between men and women, which are shaped not only by biological factors such as genetics and hormonal responses but also by societal norms and cultural environments (Feraco et al., 2024). The unique dietary behaviors and attitudes of this population make them an important focus for understanding nutrition-related issues.

The first section of the questionnaire collected general characteristics and sociodemographic information, including details about participants’ frequency of physical activity. The second section used the TOS to evaluate tendencies related to orthorexic behaviors. The third section used the FNS to measure individuals’ attitudes towards trying new foods. Anthropometric measurements were recorded based on the declarations of the individuals in the study regarding their current body weight and height. The researchers calculated the body mass index (BMI) values using the ‘kg/m^2^’ formula.

### 2.2. Measurements

#### 2.2.1. Teruel Orthorexia Scale

The Teruel Orthorexia Scale (TOS) (Barrada & Roncero, 2018) was used to evaluate the participants’ tendencies towards orthorexia. This scale consists of two dimensions: OrNe and HeOr. The OrNe subscale assesses the pathological fixation on healthy eating and its consequences, while the HeOr subscale evaluates the non-pathological tendency that also focuses on maintaining a diet consisting of healthy foods. On the Likert scale, each item was rated from zero to three. The scores of the sub-dimensions were calculated by summing the responses of the participants. The version of the TOS used was confirmed to be valid and reliable (Asarkaya & Arcan, 2021). Cronbach’s alpha coefficient of internal consistency was used to estimate the reliability of the questionnaire. The validity of the TOS was tested and was found to have a Cronbach’s alpha value of 0.883, which was considered acceptable for the TOS in the present study. In this study, the Cronbach’s alpha was 0.859 for the TOS OrNe and 0.839 for the TOS HeOr.

#### 2.2.2. The Food Neophobia Scale

The FNS was used to assess individuals’ attitudes towards novel foods (Pliner & Hobden, 1992). We used a validated and established version in Turkish that is known for its reliability (Uçar et al., 2021). The FNS is a ten-item Likert-type scale that includes items with seven response categories. Higher scores indicate a greater tendency towards FN or a reluctance to try new foods. In our study, the participants were divided into three groups according to percentiles, specifically, the 33rd and 66th percentiles (Falciglia et al., 2000; Vaarno et al., 2015). Neophilic participants scored between 10 and 34, indicating a higher willingness to accept new foods. Participants were classified into three categories based on their scores: neophilic, neutral, and neophobic. Those who scored between 35 and 41 were considered neutral in their approach to new foods. Neophobic individuals, who scored between 42 and 61, showed a marked reluctance to try new foods. The validity of the FNS was tested and was found to have a Cronbach’s alpha of 0.691, which was considered acceptable for the present study. In this study, the Cronbach’s alpha of the FNS was 0.705. This value is similar to the reliability coefficients reported in previous studies. This indicates consistency in the application of the scale across different populations.

#### 2.2.3. International Physical Activity Questionnaire

The International Physical Activity Questionnaire—Short Form (IPAQ-SF) was used in conjunction with a questionnaire item asking about the individual’s weekly amount of physical activity, with the aim of achieving at least 150 min per week. The IPAQ-SF (Craig et al., 2003) was validated in our language (Saglam et al., 2010). The responses obtained from this form classify individuals as inactive, minimally active, or highly active based on their weekly MET (metabolic equivalent of task) values. The results are interpreted according to their level of activity.

#### 2.2.4. Anthropometric Measurements

Participants’ height (m) and body weight (kg) were recorded by the researchers, and the BMI was then calculated and assessed according to the World Health Organization criteria (WHO, 2010).

### 2.3. Statistical Analysis

Statistical Package for the Social Sciences (SPSS) version 25.0 was used for data analysis. Data were expressed as means and standard deviations or frequencies and percentages. The chi-squared test for independence was used for categorical variables. The study compared numerical variables across three independent groups (neophilic, neutral, and neophobic) using the Kruskal–Wallis test, followed by the Tukey post hoc test for pairwise comparisons (Cohen, 2013). Correlations were calculated using Kendall’s tau correlation. The significance level was set at *p* < 0.05.

## 3. Results

General characteristics are shown in Table 1. The mean age of the participants was 27.77 ± 10 years. The vast majority of participants (94%) had at least a high school education. Based on the BMI classifications, the majority of participants (64.6%) were classified as having a normal weight. The study also evaluated the level of physical activity among women with IPAQ. It was found that 12.2% of the participants were inactive, 63.2% were moderately active, and 24.6% were active. In terms of FNS score classification, a significant proportion (34.2%) of participants were classified as neophilic, indicating a high willingness to try new foods. Another group (32.7%) fell into the neutral category, while a substantial proportion (33.1%) were classified as neophobic, indicating a reluctance to experiment with new foods. The mean score for OrNe was 11.45 ± 3.91, and for HeOr, it was 20.04 ± 4.31.

Table 2 presents a detailed summary of the distribution of age, anthropometric measurements, physical activity, and TOS based on the FN groups of the participants. There were no differences in age and BMI between the groups (*p* > 0.05). However, there was a difference in the distribution of BMI classifications between the three groups (*p* < 0.05). The neophobic proportion of obese participants (45.6%) was statistically significantly higher than that of neophilic participants (24.4%) (*p* < 0.05). Additionally, a significant difference was observed in the total MET scores between the neophobic and neophilic groups (*p* = 0.036). There was no difference in HeOr scores between the neutral (20.22 ± 4.24), neophobic (20.28 ± 4.57), and neophilic (19.61 ± 4.09) groups. However, the neophobic group had a higher OrNe score compared to the neophilic groups. In addition, the OrNe score of the FN groups was found to be 0.019 in the ANOVA test and partial eta squared. A significant difference with a small effect size was found in the OrNe score according to FN groups.

Table 3 shows the correlation coefficients between various variables, including age, BMI, MET, FNS, and the two subscales of the TOS related to HeOr and OrNe. Notably, FNS showed no correlation with age (r = −0.029, *p* = 0.188) or BMI (r = 0.031, *p* = 0.147). However, FNS scores were significantly correlated with physical activity (r = 0.050, *p* = 0.020), HeOr (r = 0.046, *p* = 0.038), and OrNe (r = 0.088, *p* < 0.001). The results indicate that age is positively correlated with BMI (r = 0.245, *p* < 0.001) and negatively correlated with physical activity (r = −0.100, *p* < 0.001). In addition, age is positively correlated with both HeOr (r = 0.138, *p* < 0.001) and OrNe (r = 0.087, *p* < 0.001), whereas BMI only shows a positive correlation with OrNe (r = 0.135, *p* < 0.001). Furthermore, HeOr indicated a positive correlation with OrNe (r = 0.368, *p* < 0.001), showing a link between HeOr and OrNe inclinations.

## 4. Discussion

The aim of this study was to investigate the relationship between FN and orthorexia in women. To our knowledge, this is the first study to examine the relationship between FN and two facets of orthorexia in women. Participants with OrNe scored higher on the FNS, suggesting a greater tendency towards neophobic tendencies in individuals with symptoms of OrNe. In contrast, participants with healthy orthorexic tendencies show no difference in FN.

The link between FN and orthorexia indicates that people who are more neophobic about food, meaning that they have a greater fear or reluctance to try new foods, also tend to exhibit more symptoms of OrNe. Orthorexia nervosa is characterized by an obsessive focus on healthy eating, which can lead to restrictive eating patterns. We found higher FNS scores in individuals with OrNe, suggesting that their rigid eating habits may be partly driven by a fear of unfamiliar foods. Uçar et al. (2018) showed that participants with OrNe had higher scores, indicating a stronger tendency towards neophobic tendencies. Furthermore, Hazley et al. (Hazley et al., 2022) examined the relationship between FN and dietary patterns, suggesting that FN may be associated with reduced dietary variety and quality. Our study, in line with the literature, provides evidence for the potential link between OrNe and FN, suggesting that individuals with symptoms of OrNe may be more likely to be reluctant to try new foods (Hazley et al., 2022; Uçar et al., 2018). On the other hand, participants with healthy orthorexic tendencies showed no difference in FNS scores, suggesting that the pathological aspects of OrNe are more closely related to FN than the non-pathological, health-orientated behaviors.

The prevalence of FN, or reluctance to try new foods, appears to vary between different age groups. Some studies have suggested an increase in FN among older age groups (Yodogawa et al., 2022), while others have reported a decrease in FN with increasing age (van den Heuvel et al., 2019). Although our findings did not reach statistical significance, they suggest a negative relationship between age and FN. There is a tendency for individuals to become more receptive to new foods as they age.

Many studies have investigated the relationship between age and orthorexia. Although the results are not entirely consistent, some research suggests that younger individuals may be more susceptible to orthorexic tendencies (Skella et al., 2022; Yılmaz & Dundar, 2022). However, conflicting results have been reported in the literature, and the influence of age on orthorexia, as a research topic, still lacks a clear consensus (Gkiouleka et al., 2022; Yılmaz & Dundar, 2022). We found a positive correlation between age and both HeOr and OrNe.

In line with prior research suggesting a positive association between orthorexic tendencies and higher BMI (Agopyan et al., 2019; Novara et al., 2022), we found a positive correlation between BMI and OrNe. People with higher levels of body fat, such as those who are overweight or obese, and wish to improve their physical well-being, may follow strict diets to reduce their body weight, which could increase their risk of developing orthorexic tendencies (Novara et al., 2022).

Furthermore, our results showed a positive correlation between HeOr and OrNe, suggesting a link between tendencies towards HeOr and OrNe. ‘Healthy orthorexia’ is not a medical term, but rather denotes a milder form of orthorexic tendencies, characterized by an increased focus on consuming only ‘clean’ or ‘pure’ foods. Although initially perceived as a positive mission to achieve better health, orthorexic tendencies can potentially lead to nutritional deficiencies, social isolation, and other health problems. Research has suggested that a preoccupation with healthy eating may be associated with emotional difficulties, as orthorexic tendencies have been linked to challenges in emotional well-being (Vuillier et al., 2020). It is therefore important to maintain a balanced approach to healthy eating. In summary, although the medical literature does not provide a clear definition of HeOr, we acknowledge the potential risks linked with an excessive preoccupation with healthy eating. This behavior can lead to OrNe and other health problems.

The relationship between FN and physical activity is complex and multifaceted. Some studies have suggested that there is a possible link between FN and physical activity and that regular physical activity and exercise can influence food choices and dietary behaviors (Beckford, 2018; Bellisle, 1999). However, further studies are needed to identify this relationship and the underlying mechanisms. Previous studies have shown that exercise is a factor that influences eating behavior traits associated with susceptibility to neophobia and the types of food that individuals potentially choose to consume (Guzek et al., 2018). In this study, the neophilic group had the lowest mean MET score, while the neophobic group had the highest. The study suggests a positive association between physical activity and FN, suggesting that more physically active individuals may be more wary of unfamiliar foods.

### Strengths and Limitations

The strength of this study lies in its comprehensive approach to evaluating the relationship between two dimensions of orthorexic tendencies and FN in women. The investigation of factors influencing these two eating behaviors was conducted with a substantial sample size of 985 participants. Notably, this study is the first to examine the relationship between FN and orthorexia, including its two sub-dimensions, in a large sample of women. Exploring the interplay between orthorexia, FN, physical activity, and demographic factors provides a nuanced understanding of these relationships. Our findings are expected to make a significant contribution to the current literature. The study’s commitment to advancing scientific understanding is demonstrated by the acknowledgement of complexity and the call for further research to explore underlying mechanisms and interventions.

However, it is important to consider some limitations. While a substantial sample size is crucial, the generalizability of the findings may be limited due to the homogeneity of the sample, particularly as it includes only women and primarily those with at least a high school education. Consequently, the results cannot be generalized to the male population or to women with lower levels of education. Future researchers should consider including men and diverse educational and socio-economic groups to improve the external validity of the findings. Moreover, this study did not fully account for other potential confounding variables, such as social class, cultural background, or socio-economic status, which may influence FN and orthorexic tendencies. These factors may play an essential role in shaping eating behaviors and warrant further investigation in future research. Additionally, while this study acknowledges the influence of various factors on the relationships examined, it could not comprehensively control for all possible confounders. Finally, as this is a cross-sectional study, causal relationships cannot be inferred, and longitudinal or experimental designs are recommended to explore the mechanisms underlying these associations. Nevertheless, the strengths of our study allowed it to provide valuable insights that can guide future research in understanding complex aspects of orthorexia and related traits.

## 5. Conclusions

This study offers insights into the relationships between factors and characteristics that may influence orthorexia and FN. Our findings provide a better understanding of the relationships between age, BMI, physical activity, FN, and different aspects of orthorexia. Notably, the findings suggest that individuals with OrNe have higher FN scores, reflecting a reluctance to try unfamiliar foods, while those with HeOr do not differ in their FN tendencies. This distinction emphasizes the importance of distinguishing between non-pathological and pathological eating behaviors when addressing dietary concerns. The study highlights several psychological factors that may influence eating behavior, including restrictive tendencies driven by a fear of unfamiliar foods, an increased focus on healthy eating, and the potential impact of body image concerns, as suggested by the positive correlation between OrNe and BMI. These findings highlight the need for targeted interventions that address restrictive eating behaviors and food anxiety. For instance, therapeutic approaches could focus on improving dietary flexibility, reducing rigid eating patterns, and addressing underlying psychological stressors such as perfectionism or anxiety. Interventions should also include educational strategies to promote a balanced approach to healthy eating. The findings highlight the importance of including diverse perspectives in future research, particularly studies that include male participants, broader age ranges, and individuals from different socio-economic and cultural backgrounds. Such studies would provide a more comprehensive understanding of these eating behaviors and inform the development of tailored interventions.

## Figures and Tables

**Table 1:** Characteristics of women (n: 985).

General Characteristics	All (n: 985)	
	n	(%)
**Age (years) (M ± SD)**	27.77 ± 10	
**Educational level**		
Primary school	39	4.0
Secondary school	20	2.0
High school	475	48.2
University	400	40.6
Master’s and Ph.D.	51	5.2
**Body weight (kg) (M ± SD)**	62.55 ± 12.14	
**BMI (kg/m^2^) (M ± SD)**	23.24 ± 4.57	
**BMI classification**		
Underweight	84	8.5
Normal	636	64.6
People with overweight	175	17.8
People with obesity	90	9.1
**MET (M ± SD)**	2796.62 ± 4336.11	
Inactive	120	12.2
Moderate	623	63.2
Active	242	24.6
**FNS (M ± SD)**	38.6 ± 10.22	
Neophilic	337	34.2
Neutral	322	32.7
Neophobic	326	33.1
**TOS (M ± SD)**		
TOS—Healthy orthorexia	20.04 ± 4.31	
TOS—Orthorexia nervosa	11.45 ± 3.91	

n, number of women; %, percentage of women; M, mean; SD, standard deviation; BMI, body mass index; FNS, Food Neophobia Scale; TOS, Teruel Orthorexia Scale; inactive, <300 MET; moderate, 600 MET and over; active, 3000 MET and over.

**Table 2:** Distribution of age, anthropometric measurements, physical activity, and TOS by food neophobia groups.

	Neophilic Group (n = 337)	Neutral Group (n = 322)	Neophobic Group (n = 326)	K-W Statistics	
	M ± SD	M ± SD	M ± SD		
**FNS**	27.75± 5.33 ^a^	38.69± 2.25 ^b^	48.70± 6.06 ^c^	875.429	**<0.001** ^§^
**Age (year)**	26.46 ± 9.18	27.36 ± 9.75	28.48 ± 11.00	1.761	0.414 ^§^
**BMI (kg/m^2^)**	22.85 ± 4.03	23.21± 4.33	23.67 ± 5.23	0.976	0.614 ^§^
**MET**	2528.80 ± 3396.32 ^a^	2837.62 ± 4147.67 ^a,b^	3032.96 ± 5279.88 ^b^	6.654	**0.036** **^§^**
**HeOr**	19.61 ± 4.09	20.22 ± 4.24	20.28 ± 4.57	5.664	0.059 ^§^
**OrNe**	10.76 ± 3.55 ^a^	11.52 ± 3.75 ^b^	12.07 ± 4.28 ^b^	15.788	**<0.001** ^§^
	**n (%)**	**n (%)**	**n (%)**		
**BMI Classification**					
Underweight	23 (27.4) ^a^	27 (32.1) ^a^	34 (40.5) ^a^	χ^2^: 14.799; df: 6	**0.009** ^#^
Normal	242 (38.1) ^a^	206 (32.3) ^a,b^	188 (29.6) ^b^		
People with overweight	50 (28.6) ^a^	62 (35.4) ^a^	63 (36.0) ^a^		
People with obesity	22 (24.4) ^a^	27 (30.0) ^a,b^	41 (45.6) ^b^		
**MET Classification**					
Inactive	44 (36.7)	40 (33.3)	36 (30.0)	χ^2^: 8.660; df: 4	0.061 ^#^
Moderate	228 (36.6)	200 (32.1)	195 (31.3)		
Active	65 (26.9)	82 (33.9)	95 (39.2)		

FNS, Food Neophobia Scale; BMI, body mass index; MET, metabolic equivalent of task; HeOr, healthy orthorexia; OrNe, orthorexia nervosa; ^§^ the Kruskal–Wallis test was used for the test of differences and Tukey’s post hoc test and values in the same row with different superscript letters are significantly different. Inactive, <300 MET; moderate, 600–3000 MET; active, 3000 MET and over. ^#^ The Pearson chi-squared test was used, and for row proportion comparison, the Bonferroni method was used. **Bold values denote *p* < 0.05.**

**Table 3:** Correlation coefficients between age, BMI, physical level activity, FNS, and TOS subscales.

Variables		FNS	Age	BMI	MET	HeOr	OrNe
**FNS**	**r**	1	−0.03	0.03	0.05 *	0.05 *	0.09 **
**Age**	**r**		1	0.25 **	−0.10 **	0.14 **	0.09 **
**BMI**	**r**			1	−0.01	0.03	0.14 **
**MET**	**r**				1	0.01	0.03
**HeOr**	**r**					1	0.37 **
**OrNe**	**r**						1

FNS, Food Neophobia Scale; BMI, body mass index; MET, metabolic equivalent of task; HeOr, healthy orthorexia; OrNe, orthorexia nervosa. Kendall’s Tau: * *p* < 0.05, ** *p* < 0.001.

## Data Availability

All data are available from the corresponding author upon reasonable request.
